# Digital Histopathology by Infrared Spectroscopic Imaging

**DOI:** 10.1146/annurev-anchem-101422-090956

**Published:** 2023-04-17

**Authors:** Rohit Bhargava

**Affiliations:** Department of Bioengineering; Department of Electrical and Computer Engineering; Department of Mechanical Science and Engineering; Department of Chemical and Biomolecular Engineering; Department of Chemistry; Cancer Center at Illinois; and Beckman Institute for Advanced Science and Technology, University of Illinois at Urbana-Champaign, Urbana, Illinois, USA;

**Keywords:** infrared spectroscopy, imaging, digital pathology, machine learning, deep learning, chemical imaging

## Abstract

Infrared (IR) spectroscopic imaging records spatially resolved molecular vibrational spectra, enabling a comprehensive measurement of the chemical makeup and heterogeneity of biological tissues. Combining this novel contrast mechanism in microscopy with the use of artificial intelligence can transform the practice of histopathology, which currently relies largely on human examination of morphologic patterns within stained tissue. First, this review summarizes IR imaging instrumentation especially suited to histopathology, analyses of its performance, and major trends. Second, an overview of data processing methods and application of machine learning is given, with an emphasis on the emerging use of deep learning. Third, a discussion on workflows in pathology is provided, with four categories proposed based on the complexity of methods and the analytical performance needed. Last, a set of guidelines, termed experimental and analytical specifications for spectroscopic imaging in histopathology, are proposed to help standardize the diversity of approaches in this emerging area.

## INTRODUCTION

Histopathology is a cornerstone of clinical decision-making and an important component of biomedical research in development and disease (1). Major milestones in histopathology can be directly related to the development of technology. Dating back to the seventeenth century, microscopes enabled the study of morphology at the cellular level and the structure of organs. The development of microtomes in the nineteenth century was followed by the use of paraffin wax for infiltration and support during sectioning and the use of formalin as a fixative. Automated tissue processors to replace manual processing (1940s), cryosection technologies (1950s), immunohistochemistry (1970s), and revolutions in electron microscopy (1980s) rounded out the twentieth century. Now, a transformation driven by advances in optical microscopy, information technology, computing, and data storage is underway. These developments largely compose the practices of modern-day histopathology. Digital pathology is an overarching term for the use of these modern methods that seek to increase the information content from tissues and use digitization and computerized analysis of images. While many new capabilities have been introduced, the key processes in pathology for acquiring data—sample preparation, staining, and optical microscopy—have remained remarkably consistent for over 125 years. In this review, I focus on infrared (IR) spectroscopic imaging as a source of new contrast and its potential role in enabling digital pathology.

IR spectroscopy has been used for biological analyses for over 125 years as well. Average tissue spectra get confounded by cellular composition or other factors (2), and the need for spatially resolved IR spectra has been recognized since the 1950s (3). However, few spatially resolved measurements could be conducted for several decades due to limitations of instruments and data interpretation. The intervening invention of the fast Fourier transform (FT), development of FT-IR spectrometers, and coupling of spectrometers to microscopes coincided with increased computational and data handling capability. Point-scanning FT-IR microscopes reignited an interest in histopathology (4, 5), and the use of pattern recognition enabled correlations of spectra to histopathologic identity in a map (6, 7). Other than using synchrotrons as sources (8), however, the data had to be acquired from regions that were tens of micrometers wide, from a few hundred spectra per sample, and from a few tens of samples at best, often necessitating spectral analysis from tissue units rather than from small pixels (9). The development of FT-IR imaging using array detectors led to images that resembled optical microscopy images and numerous applications to studying tissue (10–12). A turning point for histopathology was the use of small (~5 μm) pixels in large spatial scans that sought to produce images resembling those in pathology, proof of concept with statistically large and diverse samples using tissue microarrays, and design of fast machine learning (ML) workflows specifically for this data type (13). Millions of pixels per sample, diverse data sets from tens to hundreds of samples, and ML workflows with multiple steps to provide rigorous training and validation are generally recognized as the contemporary approach to tissue histopathology studies. Reviews (14–20) and compilations (21–23) that cover the evolution of technology (24) and progress toward translation (25) describe the development arc of the field until a few years ago. Here, the focus is on describing the main analytical considerations in technology and computational methods, application themes, design of workflows, and emerging directions.

## THE POTENTIAL ROLE OF INFRARED IMAGING IN PATHOLOGY

The primary role of histopathologic analyses is to provide powerful visual evidence of altered tissue structure and, less frequently, of molecular composition. To appreciate the role of IR imaging, key trends that pave the way for utility of this technique are summarized next. Histopathologic images have traditionally provided qualitative evidence that was interpreted in terms of broad guidelines that characterize disease severity or development stage, with common numbers often serving as surrogates for outcome of the disease. For example, prostate carcinomas are classified (26) in the Gleason grading system using a five-point scale (grades 1–5), with the higher number indicating a more lethal phenotype. However, this broad classification does not adequately serve an individual patient (27). These measures were designed to be not too complex so that they are interpretable by humans, are broadly recognizable in most cases, and serve to educate and train clinicians and researchers to a common standard. Understanding the full complement of the disease and precision medicine were not the goals then, as they are today, in guiding patient choices and therapeutic strategies (28). Thus, there is an unmet need for new measures that go beyond established pathology practice. In addition to molecular markers, awareness of the importance of diversity in spatial expression of molecular markers has grown (29) and knowledge of the involvement of the microenvironment in disease progression has greatly increased (30–32). Measures of disease severity that focused largely on a single cell type are now recognized as suboptimal in understanding and prognostication (33). In this milieu, two major directions have emerged: first, the use of ML to better interpret tissue structure and disease state, resulting in the emergence of digital pathology (34, 35), and second, increased molecular measurement capability to augment morphologic analyses (36). Driving both directions are the explosive rise in computing capability, including faster processors, cloud computing, and use of graphical processing units or cluster computing; the ready availability of massive digital storage at ever-decreasing costs; increasingly sophisticated and capable ML tools, including deep learning (DL); and a broad acceptance of digital methods in pathology. While molecular methods were hypothesized to transform pathology with the dawn of the genomic age, trends in computing have ensured that optical microscopy, stains, and morphologic evaluation remain preeminent, with molecular expressions used largely for confirmatory tests (37).

### IR Imaging Offers an Information-Rich and Streamlined Histopathologic Workflow that Combines Morphologic and Molecular Domains

**Figure 1:**
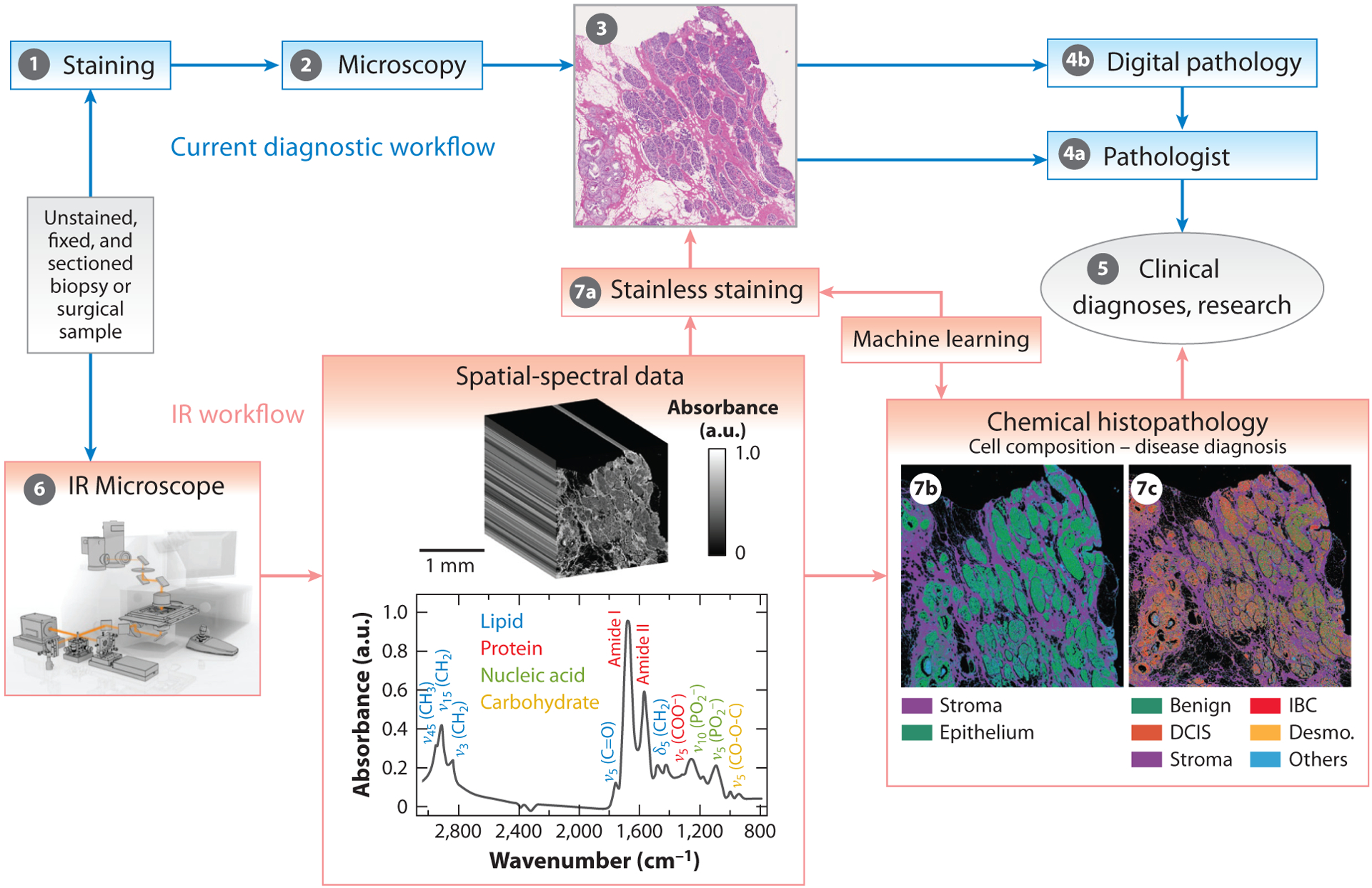
Current histopathologic evaluation process and the proposed process based on IR imaging. Tissue is first obtained, fixed, and embedded in a cutting medium, and a thin section is obtained on a slide as the sample. (Step 1) The sample is stained, commonly with H&E. (Step 2) Optical microscopy is the mainstay for visualizing tissue morphology to diagnose disease or for research assessments, providing (Step 3) images in which contrast is enhanced by the stain. (Step 4a) Images are assessed by a pathologist. (Step 4b) Digital pathology, wherein computer algorithms are used to assist the pathologist by quantitative morphological information, is an emerging aid. (Step 5) Tissue evaluations can then provide input for clinical diagnoses or research insights. In contrast, steps 6 and 7 show the workflow possible with IR imaging. (Step 6) An IR microscope, providing the benefits of microscopy and spectroscopy, integrally uses a computer because of the large volume of data and impracticality of manual interpretation. The data have two spatial dimensions, similar to optical microscopy, but a much larger spectral dimension. Machine learning is applied in two important areas: (Step 7a) stainless staining, to reproduce the stained images commonly used in pathology, and (Steps 7b and 7c) chemical histopathology, to gain novel information over present methods and visualization that eliminates the need for painstaking examination of tissues. (Step 7b) Segmentation of tissue into epithelial cells and other components (collectively, the stroma), and (Step 7c) comprehensive examination of breast tissue for disease and stromal reaction. In Step 6, the image is adapted from Reference 24 and the spectrum is adapted from Reference 38. Abbreviations: DCIS, ductal carcinoma in situ; Desmo., desmoplastic stroma; H&E, hematoxylin and eosin; IBC, invasive breast cancer; IR, infrared.

A comparison of the current histopathology workflow and possibilities with IR imaging is shown in Figure 1. The multistep nature of the illustrated current workflow (see Steps 1–5) requires physical transport of the sample, synchronization of reagents, human resources and measurement tools, and an application of digital methods. In contrast, IR measurement (Step 6) and application of ML (Steps 7a–c) form a compact, two-step workflow to reduce effort and yield fit-for-purpose visualizations that are more informative. Since the sample is unstained, one direction has been to generate stainless images that resemble pathology images (Step 7a). A second direction is to afford rapid visualization of composition and disease, for example, allowing for identification of epithelial cells in which most cancers occur (Step 7b) and using a different ML model (Step 7c) to identify disease in an accurate, objective, and automated manner. The combination of IR imaging and AI promises to provide to the pathologist both old and new information arising from the native chemical composition of the material. It can save experts time and effort and provide benefits in time-critical settings such as intraoperative venues. Because it does not require a reagent, IR imaging can save on material costs and on process and labor costs needed for staining, as well as avoid variability arising from technician, sample batch, and specific protocols to provide more consistent data. Since IR imaging is nonperturbative to the sample, the tissue is preserved for other analyses. Because it is applicable to fresh, frozen, or archival tissue, the same method can be used, and information from one type of sample can be transferred to another type with ML. Finally, significantly more information is obtainable from within the same spatial context in one measurement. Color-coded images, as opposed to the limited palette of dyes conventionally used, make interpretation easy and provide an opportunity to study each cellular component individually or in the spatial-chemical context of other components. The goal of spectral analyses for pathology is also unlike that of conventional molecular spectroscopy, in which the spectrum is related to the identity and molecular structure of a single chemical. Instead, ML relates tissue composition to its functional end points (e.g., cell identity, physiology, or disease). While these benefits contrast IR imaging with current microscopy and spectroscopy, it can also be compared with other techniques’ benefits and drawbacks.

#### IR imaging is a unique and complementary approach compared with other optical techniques.

A brief description of the context of other approaches relevant to pathology is useful for understanding the potential developmental directions for IR imaging. Optical microscopy continues to be a mainstay of imaging; recent developments include compact, lens-free systems (39) and ptychography (40), which IR imaging at present cannot beat in terms of cost and speed. Microscopy with surface excitation offers a slide-free approach with high-resolution imaging (41). Likewise, quantitative phase imaging (42, 43) can rapidly define fine structures and orientation in thin sections without the use of dyes. Light sheet microscopy (44) can provide volumetric images (45, 46) that open up the third spatial dimension for histopathology, and tissue clearing and virtual 3D reconstructions are active areas of investigation (47). Although these techniques provide new spatial capabilities, they do not have chemical specificity. While autofluorescence imaging is simpler and of higher resolution (48), IR data have much greater native chemical detail and the chance to confound algorithms is consequently lower. IR spectroscopy is sensitive (38) to all biological building blocks (i.e., proteins, nucleic acids, lipids, carbohydrates, and other small molecules), presenting the strongest molecularly sensitive signals of any optical method, as electromagnetic frequencies are directly resonant with molecular vibrational frequencies. The strong absorption provides a quantitative measure of a species’ concentration and the environment of vibrational modes is manifest in the features of the absorption peak. Raman spectroscopy offers a potentially similar benefit, but because the Raman effect is much weaker, it requires either fast scanning approaches or more expensive nonlinear Raman imaging (49, 50). Raman imaging–based histology (51, 52) can probe within the body (53, 54) and two-photon approaches provide exquisite spatial detail (55–58), but neither has yet approached the level of chemical detail and spatial coverage of IR imaging. The broad field of nonlinear multiphoton microscopy is not covered here but offers a large menu of opportunities that can interrogate physiology, local organization, and chemical detail in living tissue and in real time. In general, these approaches are based on complicated and expensive instruments compared with IR imaging and are more suited to in vivo imaging rather than the archival pathology workflow in Figure 1, wherein thin sections, fixation, and the need for chemical detail beyond morphology reduce many of their advantages. Spatial transcriptomics (59) and molecular ion beam (60) approaches can provide ultrahigh spatial and molecular detail but at considerably higher costs and with greater complexity in interpreting the results. Similarly, mass spectrometry (61) can provide high molecular detail but is destructive and slower and pixel sizes are larger. Altogether, IR imaging presents a balanced approach to rapid spatial coverage with high chemical sensitivity with sample handling that is compatible with pathology. The most crucial need in IR imaging today is to harness these advantages and devise solutions that lead to higher-quality, accessible, and equitable care at lower cost. The analytical workflow—measurement technology, data handling, computerized interpretation, validation, and presentation of results—is critical toward that goal. Since data handling and obtaining results are determined largely by the technology used, I focus first on instrumentation.

## INFRARED SPECTROSCOPIC IMAGING TECHNOLOGY

Recording data of spatial-spectral characteristics that allow problems to be solved, with sufficient robustness and accuracy and in acceptable measurement and analysis times, is the central aim of IR methods for pathology. Thus, understanding the performance of measurement technology is critical (20, 24, 62, 63). The most common approach is to measure IR light transmitted through the sample to record absorbance.

### Established Technologies

The two broad classes of instrumentation measure either large contiguous regions of the spectrum (e.g., FT-IR spectroscopy) or a few features of interest [discrete frequency IR (DFIR)] for utility in histopathology (for a comparison, see Table 1).

#### FT-IR spectroscopic imaging is well-established, high-fidelity, and validated technology for histopathologic studies, but is slow.

**Figure 2:**
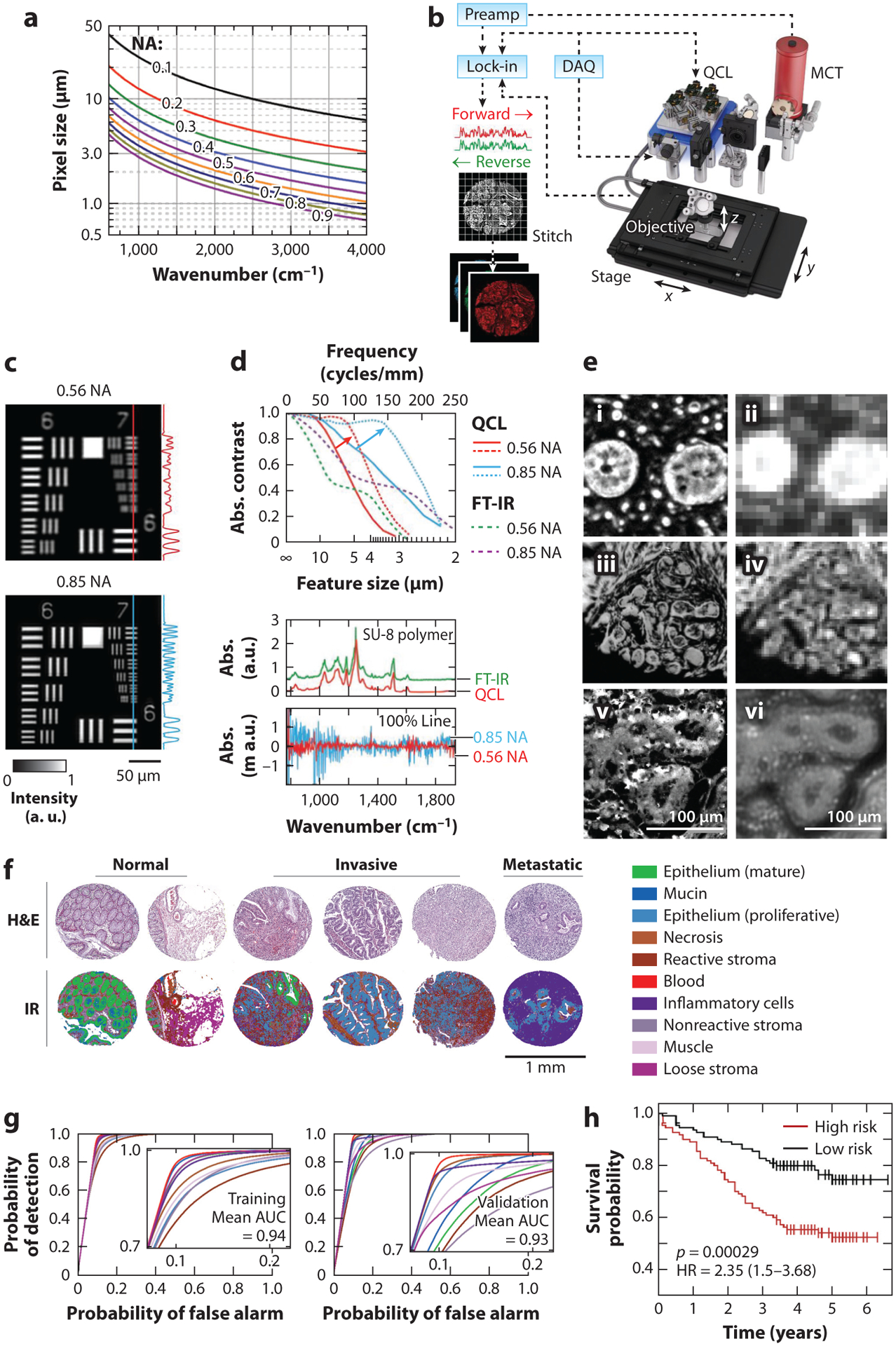
IR spectroscopic imaging measurements and use. (*a*) Theoretical prediction of the smallest pixel size to achieve the highest spatial fidelity. Panel *a* adapted from Reference 65. The graph provides a guide to designing IR imaging systems. (*b*) A custom-built DFIR imaging system, showing the essential components of an IR imaging system. (*c*) Evaluation of the spatial quality of the imaging system with two different objective lenses. (*d*) Spatial (*top*) and spectral (*bottom*) performance can be quantified. Panels *b*–*d* adapted from Reference 72. (*e*) Augmented performance of optimal optical design (*i*) compared with that of commercial systems (*ii*). Use of a solid immersion lens can increase the quality of images, providing higher resolution (*iii*) than standard images (*iv*). Using a hybrid microscopy format can provide optical microscopy resolution (*v*) compared with the optimal all-IR resolution (*vi*). Panel *e*, subpanels *v* and *vi*, adapted from Reference 73. ( *f* ) FT-IR imaging data provide high-quality spectral and spatial information that can provide color-coded pictures of the colon tumor microenvironment. (*g*) Statistical accuracy of detecting tumor and microenvironmental cells. (*h*) Prediction of risk for moderate-grade tumors. Panels *f*–*h* adapted from Reference 68. Abbreviations: Abs., absorbance; AUC, area under the curve; DAQ, data acquisition; DFIR, discrete frequency IR; FT-IR, Fourier transform–IR; H&E, hematoxylin and eosin; HR, hazard ratio; IR, infrared; MCT, mercury-cadmium-telluride; NA, numerical aperture; Preamp.; pre-amplifier; QCL, quantum cascade laser.

The advantages of interferometry (64) for IR imaging are well described, and rigorous analysis (65) of the optical imaging system acting as a filter for spatial frequencies (66) has now provided a standard for high-definition images. Interferometry and the use of multichannel detectors provide high spatial and spectral fidelity data that are the gold standard for comparisons in developing new instruments and are useful for both discovery and initial exploration for histopathology. Figure 2a–d shows the theoretical limits of performance and the current state of the art. Changing the pixel sampling density, numerical aperture (NA), or detection wavelength can improve spatial quality (Figure 2e). However, the weak thermal source and relatively noisy and slow detectors remain a limitation for whole slide imaging (WSI) because of the need to signal average to obtain signal-to-noise ratios (SNRs) sufficient for tissue classification (67). With sufficiently large measurement times, FT-IR imaging is an exceptional technology by which to examine subtle changes in tissue and to use the data for high-quality predictions, as shown in Figure 2f (68). Emerging hardware developments promise to raise performance with broadband, supercontinuum laser sources (69, 70) and digital IR focal plane arrays (71). However, once analyses are conducted, typically only a few spectral features are required for many histopathologic examinations (13). The acquisition of large FT data sets may be expensive in terms of time, analysis, and data storage. Further, the availability of brighter, narrowband, and shot noise–limited sources argues against interferometry, which is ideally suited to weaker broadband sources. Regardless, FT-IR imaging, with its incoherent thermal source and ability to cover the mid-IR region, remains the research method of choice to assess tissues for their chemical composition.

#### QCLs have enabled DFIR imaging and have greatly reduced times for histologic images.

With the realization that histologic analyses could be sped up by acquiring only relevant data, DFIR imaging was first introduced with narrowband filters (74, 75) as a source. This alternative has been variously termed discrete chemical imaging, bond-selective imaging, and vibrational mode imaging. The commercial availability of quantum cascade lasers (QCLs) (76, 77) as narrowband-emitting but wide-bandwidth tunable laser sources soon made them the source of choice for DFIR imaging. Though optical parameteric oscillator–based laser systems (78) are comparable spectral sources (and can provide some spectral coverage that QCLs cannot), the compact packaging, availability over the important fingerprint spectral region, and relatively simpler operation have made QCLs a widespread choice for IR imaging. The first IR imaging systems with QCLs (79) suffered from laser noise and instability, speckle, and suboptimal integration with existing microscopes.

Although speckle from beam coherence was mitigated with a rotating diffuser, the recorded data suffered from poor illumination and noise characteristics. Optical configurations have improved considerably since then and two directions have emerged: use of a widefield setup with a focal plane array detector (80) and confocal laser scanning (72). Widefield systems first utilized small, cooled detectors (128 × 128 pixels) for high SNR, but uncooled detection (81, 82) is also effective for histologic applications (83–85) and makes instrumentation simpler and cheaper while rapidly providing large images due to their larger format (typically 480 × 640 pixels). Widefield techniques generally use one or more optimally designed diffusers (86, 87), time delay and integration (88), spatial averaging, and spectral dithering to reduce speckle; however, the uncertainty introduced by speckles in quantifying spectra for histopathology has not been systematically examined. A net result is that large areas can be surveyed but the time to acquire high-SNR data increases while images are typically limited to ∼5 × 5 μm pixels. Laser scanning illuminates a single point at a time and largely avoids the complications of speckle while obtaining an SNR suitable for histopathology (89, 90), providing instrumentation whose spectral SNR and spatial quality are close to theoretical limits (Figure 2b–d). The downsides are a relatively lower pixel rate and the need to design microscopes that have large fields of view (to avoid frequent stage movements).

### Emerging Techniques and Trends

The diversity and capability of instruments utilizing IR lasers are likely to increase greatly, and the capacity for translational histologic studies will dramatically improve (91). Some notable techniques related to the emergence of DFIR microscopy and lasers are discussed below.

#### AFM-IR can provide ultrastructural resolution.

The combination of atomic force microscopy (AFM) imaging and IR (92) excitation promises ultrastructural (currently as small as tens of nanometers) resolution (93) that can be important for identifying subcellular domains in pathology. Though there are few occasions that require resolution this fine in clinical diagnostics, such a tool could prove valuable for research and for understanding the signals recorded in far-field modalities. Whereas AFM-IR has provided exquisite images of nanoscale materials, including plasmonic effects, detailed images of cells and tissues have been less common (94). Thicker samples imply that the resolution gets blurred; a signal deficiency for thinner samples is often a limit. Moreover, mechanical coupling (95) between the sample and the tip may affect or even dominate the signal. As opposed to other techniques, the mechanical detector involves a physical movement that can be susceptible to noise and other forces and measurements of small volumes are challenging. A technique of null-deflection that allows detailed nanoscale maps of cells with contact-mode AFM was proposed (96). The interactions and contact between the tip and the sample may cause shear deformation of the sample as the tip is scanned and may move small samples. Tapping-mode AFM-IR (and other new variants) was developed as an alternative and is being used for biological imaging (97), but its applicability may be limited for histopathology because it is significantly surface sensitive and does not sample the bulk. While applications in this area are still emerging and there are no obvious opportunities for direct application to histopathology, the availability of data from subcellular domains promises to greatly increase our understanding of microscopy data.

#### Hybrid optical microscopes (light-IR, fluorescence-IR, and Raman-IR) seek to bridge the resolution gap while adding more information.

Absorption of light causes a rise in temperature and a concomitant change in physical and optical properties. Using these photothermal changes for microscopy has become an active area of investigation. Recent reviews summarize the development and current state of the art of these technologies (98, 99). While subcellular detail can be obtained for small regions (100–103), widefield approaches are promising for routine histopathology (73, 104, 105). IR–optical hybrid (IR-OH) microscopy, in particular, provided large-field-of-view data that could be virtually stained and that resembled stained images (73). These microscopies offer an exciting new direction in IR imaging, wherein numerous contrasts can be harnessed and IR images can be obtained that are comparable in resolution to their optical counterparts of much shorter wavelengths, instrumentation can become more compatible with other microscopes, and intact specimens can be imaged. These techniques also offer significant potential to develop theory models that link observed changes to spectral content and to understand the limits of performance. New applications are already emerging in which the spatial resolution is proving to be of value to histopathologic analyses (106, 107). Use of a confocal pulsed UV laser beam to detect the temperature rise upon absorption is a novel optic-acoustic measurement scheme (108) that promises histologic imaging in intact, living samples.

#### Linear and circular polarization measurements provide additional value.

Most applications have used isotropic absorbance of samples; however, many biological systems have orientation that is related to function (e.g., collagen fiber bundles) and most molecules of interest are chiral. Linear and circular dichroism can probe these properties of tissues as new information linked to disease progression. FT-IR imaging was too slow for rapid measurements, due to both the need to scan multiple optical states and the loss of light because of optics needed for polarization measurements. New microscopy designs measure vibrational circular dichroism, allowing for measurement of spectra from small volumes and maps of chirality (109, 110). More detailed orientation maps for tissue (111, 112) that use linear dichroism imaging (113, 114) have been reported. These initial demonstrations are encouraging and are likely to spur the development of a full theoretical treatment for polarization-sensitive IR microscopy. With improved understanding, new measurement approaches and analyses of polarized IR microscopy data may contribute a new avenue to histopathology studies.

#### Postsample, wavelength-mixing innovations seek to harness the high SNR, large format, and speed capabilities of shorter-wavelength detectors.

IR array detector technology continues to improve but lags visible detectors in efficiency, cost, and size. An important bridge is to use upconversion techniques (115) wherein mixing with a stable narrowband laser (e.g., at 1,064 nm) via a nonlinear crystal conserves the IR image but now makes it detectable by sensitive detectors at shorter (visible) wavelengths. Recent progress has driven conversion efficiencies into a substantial fraction of incident photons (>0.2) and the low noise of the visible detector provides data of high SNR. The size of IR regions (which are ~20-fold larger than visible regions), accessing the broad bandwidth of spectra, speed, and resolution (still determined by IR illumination) are all factors for improvement. The use of nondegenerate two-photon absorption (116) with an InGaAs camera (117) has led to the fastest recorded (1 megapixel, 100 fps) IR images. The development of this technology promises to bring the benefits of visible-IR/near-IR detectors to the mid-IR regime.

#### Nonlinear imaging techniques promise measurements with special properties.

Using third-order sum frequency generation (TSFG) in a laser-scanning microscope (118), one can probe an IR-driven coherence via a two-photon hyper-Raman interaction, producing an image in the visible part of the spectrum. This four-wave mixing technique is sensitive to the *χ*^(3)^ properties of the sample and is not limited to certain symmetries such as second harmonic generation. TSFG signals carry vibrationally resonant and nonresonant contributions and high-speed modulation can potentially eliminate significant noise, but TSFG signals are limited by the bandwidth of visible light used to image relatively small IR spectral regions.

#### Both computational and physical methods are being developed to identify specific molecular species.

Whereas IR data rely on the spectrum at every pixel to act as a barcode of the physiologic or disease state, conventional pathology is more focused on specific molecular expression. Two approaches bridge this gap. The first is stainless staining (119), in which IR data can be related to molecular expression using ML. As shown in Figure 1, IR data can be correlated to the common hematoxylin and eosin (H&E) stain and generate images that resemble clinical images. IR-OH microscopy (73) provides pixel sizes directly matched to optical microscopy and a faithful representation of H&E images, even for relatively low-quality spectral data, likely because H&E is a rather simple stain and correlates with global protein and nuclear expression. More chemically specific stains to identify collagen (such as picrosirius red) can also be reproduced well by stainless staining (120). While highly effective for more abundant chemicals and when spectral absorbance correlates with large chemical differences between cells, results from stainless staining for small, subtle changes in molecular expression are likely to get confounded. Specificity of molecular expression is a major concern, as the overall chemical state of a cell may not be reflective of a single species. Typically, a cascaded approach is used to reduce the risk of nonspecific expression. For example, cytokeratin is present only in epithelial cells; hence, a first step was to segment tissue into epithelial cells and then analyze epithelial spectra to predict the expression level (119). Similarly, a five-step scheme was used (121) in a carefully designed study that could achieve not only histologic and disease classification but also a sensitivity and specificity of 95% for each of the three mutations important in lung cancer. The second approach to bridge the molecular and spectral domains is to use IR tags or labels, wherein an IR reporter is attached to a specific molecule(s) of interest and the amplification of a signal can be achieved by using probes with IR absorption that can be tuned to be high and away from common tissues’ vibrational modes (122, 123). Finally, photothermal approaches using fluorescent reporters or native species (124, 125) combine the best benefits of both microscopies, providing sensitive absorption and localized molecular reporting.

## DATA HANDLING AND MACHINE LEARNING

IR spectra and their mathematical analyses have a deeply intertwined history. Methods developed over decades for high-quality, full spectra, however, are unsuited for imaging data. First, spatial-spectral correlations are significant and must be considered. Second, spectrum-by-spectrum computational operations (e.g., curve fitting) do not scale well with the number of pixels in histology imaging data. Third, the high dimensionality of data; the inherent complexity of tissue; and the variability arising from clinical, sample preparation, population diversity, and disease variables need to be considered. Fourth, biological knowledge or domain expertise from pathology is difficult to incorporate. Fifth, data from imaging systems have lower SNRs than do bulk spectrometers, and the image formation process may introduce sample-structure-dependent effects.

**Figure 3:**
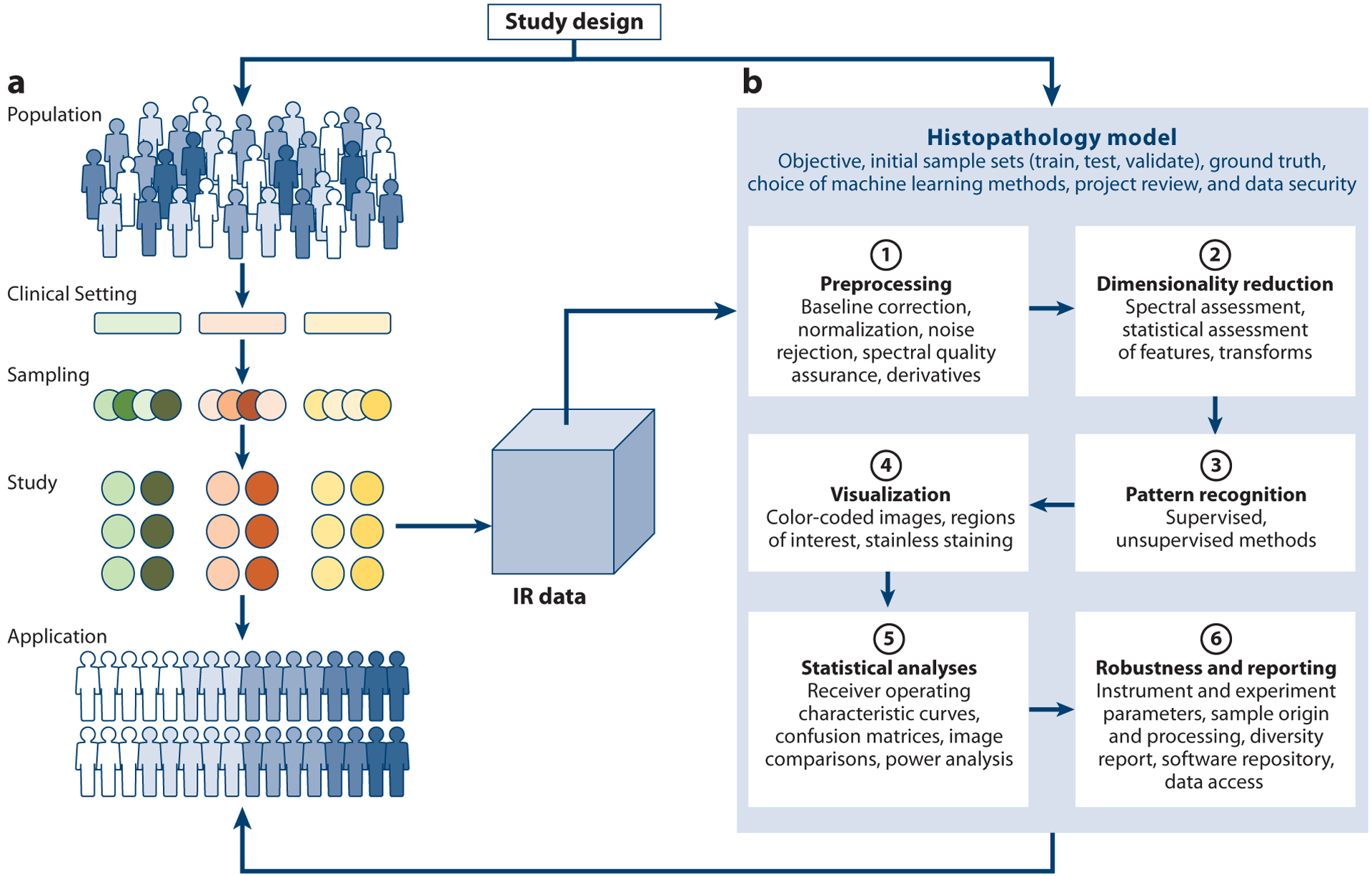
Study design and data workflows in infrared (IR)-based pathology. (*a*) General idea of a study design. A population of specific disease states is identified from several clinical systems for the study to assure a diversity of patients and practices. Representative cases for IR imaging are identified and tissue samples are prepared, often building in sampling redundancy (i.e., sampling from the same patients), use of matched cases (from the same patient or matching for known variables), and high statistical numbers. From each sample used in the study, an IR imaging data set is obtained. (*b*) A computational pipeline is then devised to assess the use of a histopathology model along with analytical parameters to process the data and extract information, with statistical validation. Multistep processing workflows are designed for specific cases as needed and benefit from the opportunity to include substantial spectroscopy and pathology knowledge. Colors of the population indicate the natural variation as well as variation due to disease, while variation introduced by clinical settings and sampling is indicated by additional colors.

**Figure 4:**
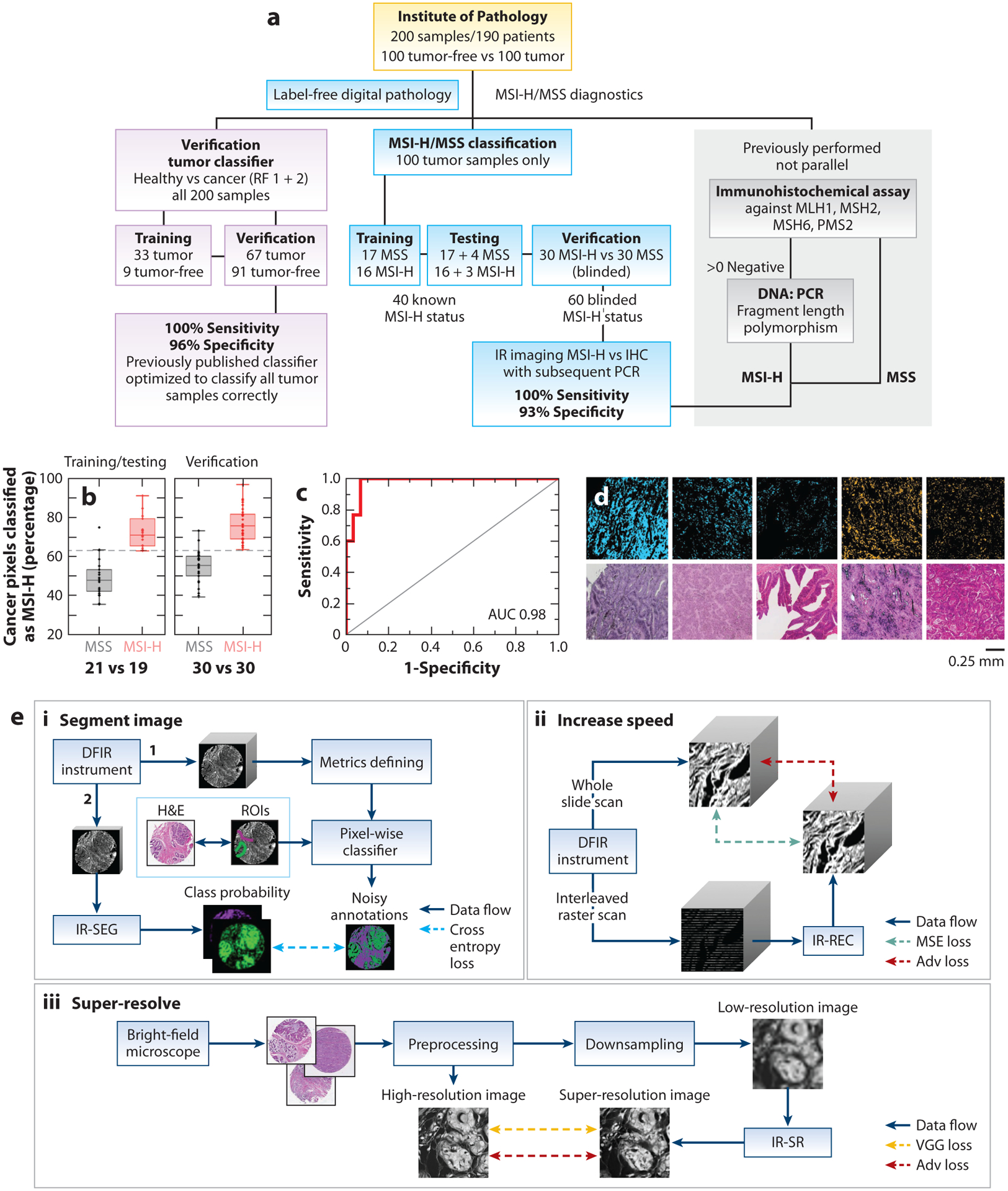
Designer workflows and use of deep learning for histopathology. Carefully crafted studies focus on a specific problem with custom design and statistical measures that relate back to the imaging data. (*a*) A well-crafted workflow for classification clearly listing the study design and experimental steps, including statistically valid results with independent validation. One workflow distinguishes healthy from cancer samples (*purple boxes*). The middle workflow (*blue boxes*) distinguishes MSS patients from MSI-H ones. The IR analyses are validated by clinical molecular analyses (*gray boxes*, *right*). (*b*) Explicit differences between MSS and MSI-H cohorts for training/testing (40 samples, 21 MSS and 19 MSI-H) and for verification (60 samples). The gray dashed line represents a threshold (63%) that segments the two groups. (*c*) Receiver operating curve with AUC demonstrating high accuracy. (*d*) Projection of classification back to images (*top*; IR images in which MSS is indicated by blue and MSI-H by orange) and comparison with H&E images (*bottom*), demonstrating the ease of conveying information with IR-classified images. Panels *a–d* adapted from Reference 83, with permission from the authors. (*e*) Deep learning can be used to (*i*) segment images in one step, (*ii*) increase speed by estimating missing data, and (*iii*) use multimodal information to super-resolve images using data-driven algorithms. Panel *e* adapted from Reference 129; copyright 2021 Elsevier. Abbreviations: Adv, adversarial; AUC, area under the curve; DFIR, discrete frequency IR; H&E, hematoxylin and eosin; IHC, immunohistochemistry; IR, infrared; IR-REC, IR-reconstruction; IR-SEG, IR-segmentation; IR-SR, IR super-resolution; MSE, mean square error; MSI-H, high microsatellite instable; MSS, microsatellite stable; PCR, polymerase chain reaction; RF, random forest; ROI, region of interest; VGG, visual geometry group.

Finally, quality control and proof of concept are often carried out in the limit of small numbers of samples, which generally require careful assessment and development of appropriate ground truths. For example, ground truth in traditional spectral analyses may be a concentration of a specific species, which likely has a unique spectrum that is conserved in all formulations, whereas pathologic ground truth may be disease grade, with numerous chemical manifestations and large diversity. Consequently, workflows must be creatively and carefully designed to be tolerant of diversity (not just noise) in the data, uncertain ground truths, a range of possibilities in output with a confidence rating, robustness in application, and stability with respect to small changes in conditions. This analytical complexity, illustrated in Figure 3a, implies that study design and data handling must be closely related in light of variances from these factors (126). These considerations also mandate that developing an appropriate strategy is critical for the desired diagnostic test (vide infra). For most studies, the data processing workflow consists of several steps that are remarkably similar, with multiple, sequential operations (127) that require spectroscopy and pathology knowledge to ensure success (Figure 3b). One example of this success is depicted in Figure 4a, wherein a purpose-driven workflow yields clinically useful results after training (of the algorithm to predict desired outputs) and validation (of the results). Given the increasing role of complex ML, a middle step of testing (the robustness and optimal nature of parameters) is often now included when developing workflows. While training and testing can be accomplished on the same data set and may include strategies such as dividing a single data set multiple times into parts to be used for testing and training (cross-validation), validation studies should be undertaken with an independent data set whenever possible. Given the largely statistical nature of the analysis, careful study and algorithm design, as well as appropriate sample size and diversity, are bulwarks against the results being influenced by chance or bias. Due to space limitations, this review does not discuss the major techniques and operations; numerous sources describe hyperspectral image analyses, ML, and various IR pathology–related aspects (128). Given the recent emergence of DL, I briefly describe its potential for IR histopathology.

### Deep Learning

Details on DL fundamentals, capabilities, and applications can be found elsewhere (130) and in a review of spectroscopic imaging applications (131). The transformative development for IR histopathology is that DL provides an opportunity to change the development of protocols and validation. First, it can potentially reduce the burden on the spectroscopist to develop hand-crafted workflows, since its multiscale examination of the data yields automated predictive relationships. Second, significantly more powerful analyses of the spectral, spatial, spectral-spatial, and multimodal data will be possible. At the same time, however, interpretation of these analyses and assurance that they are not brittle in light of small analytical modifications will be major challenges. Third, the burden of computational design and progress is being borne by a much larger community, ensuring that extensive programming knowledge is not needed and that specialized, more powerful methods that are fit for purpose are becoming increasingly available. Fourth, computational power and storage power, including architectures devoted to DL, are rapidly increasing. These are particularly advantageous for IR imaging of large data sets and the complexity of biological samples. Fifth, advances in DL are also driving an increased realism in images and interactive augmented reality capabilities. The practice of pathology has been rich with both human interpretation and use of technology—the combination of powerful analyses and realistic, information-rich visualizations will be a useful direction. Finally, DL integrated with instrumentation can speed up data acquisition and alter the trade-offs in measurements that arise from physical factors alone, transitioning computational analyses from a postacquisition analytical role to an integral one in the entire measurement chain. Details on some of the major trends that are becoming apparent or will soon be significant directions are discussed next.

#### The limits of conventional physical trade-offs can be transformed by DL computational estimation.

Figure 4e shows three examples of how data may be augmented (129). First, histopathologic information may be obtained by DL with far greater ability and less data compared with conventional methods, including such steps as variable selection, scattering-induced baseline estimation, and noise rejection. Second, DL promises to complete the acquired imaging data set when fewer pixels than needed are acquired, when the SNR of acquired data is less than needed for accuracy dictated by the task, or when pixel density is lower than desired. The ability to estimate missing data comes from the powerful correlations inherent in the latent space of the algorithm from many observations and can improve continually with more data. However, two considerations are critical to understand when applying such techniques. First, the confidence in interpolated or estimated data cannot be more than that obtained from the measurement itself.

Any such predictions necessarily draw from past behavior (e.g., database of spectra or images) or additional information (e.g., using visible images to infer IR spectral data) that may not add more certainty. Indeed, measurement is a form of estimation, often with well-defined uncertainty. Using ML to estimate gaps in capability or measurements is also an estimation, albeit with uncertainty that comes from the data, from the examples used for learning, and from the opacity of the predictive method itself. Second, the use of ML techniques to aid measurements must be conducted with the realization of the consequences of potential hallucinations or errors, as the use of these powerful techniques may lull the practitioner into disregarding notions of limits of their performance. This makes it essential to understand the information content of the data and to carefully apply ML techniques to enhance capability within these limits. For example, the resolution in IR imaging is determined by the optical system (optics, coherence of illumination, aberrations), the sample (geometry, separation from the analytical background, prior knowledge), and noise. The minimum resolvable distance, *d*, is often described by$d=f_{r}\frac{\lambda }{\text{NA}}$,where *f*_*r*_ ranges from 0.4 to 0.61 for different criteria. However, these relationships are not derived from a specific rule other than separability of two identical objects. IR imaging follows the same rules for identical point absorbers; however, it is often the case that there are spectral differences in IR imaging between neighboring point absorbers. To understand the information limits of such data, a relationship (132) for far-field IR microscopes has been developed:

$$\hat{\text{d}}=\frac{\lambda }{\text{NA}}\frac{1}{{\text{log}}_{2}\sqrt{1+\text{SNR}\left(1+4\Delta^{2}\right)}}.$$

The equation shows the familiar dependence on wavelength and NA but now explicitly describes the relationship between SNR and material properties (Δ, or spectral distance) as well. This relationship is of the same simple form as common microscopy resolution criteria but is rigorously accurate in terms of information content. Deconvolution methods, for example, should not attempt pixels finer than dictated by the data. However, DL offers the intriguing possibility of adding new, orthogonal information. As shown in Figure 4e, the inclusion of features from a visible microscopy image potentially provides textures beyond these limits. As seen from this example, DL has the potential to redefine the capability of histologic segmentation by reducing the need for data acquisition, to reduce experimental parameter range by estimation, and to overcome information content limits by injecting new information.

#### Highly accurate histopathology with IR contributions to multimodal histopathology will become more useful and common.

Carefully crafted workflows have dominated studies thus far and have moved accuracies from approximately 70% to more than 90%. DL protocols now promise nearly perfect histologic recognition due to their complexity, inclusion of spatial-spectral textures, and use of large training data. With fast imaging, it can be reasonably expected that high-accuracy studies of different tissues will result for a wide range of pathologies. Caution is needed when evaluating uncommon occurrences, and the lack of accuracy will likely arise from these outliers that were not in the training database. In an interesting crossover, IR data were used to generate deepfake H&E images (133) that were practically indistinguishable from real images by board-certified pathologists. By subtle changes in the IR map, new H&E pathologies that are not commonly encountered could be generated, thus increasing the predictive ability of morphology-based algorithms. This integration will likely be driven further as multimodal information becomes more common in pathology.

#### Increasing measurement capability will provide more data to address confounding variables.

The combination of different models, effect sizes, and data used, including the numbers of tissue samples, makes it challenging to provide a ready guideline for a satisfactorily robust algorithm (134). A general consensus is that modern imaging and tissue microarrays allow data to be recorded from hundreds of patients’ (relatively small) samples. Since spectral features are central to DFIR imaging, the desire to reduce computational effort and data size while maintaining high accuracy is important (135). This interplay of sufficient samples to make workflows robust yet minimize the effort for data acquisition will be a major theme. Samples from at least 50 patients, with multiple samples per patient, is a reasonable number to obtain initial data and estimate accuracy and robustness for most protocols. Extensive, independent validation with sample numbers and statistics dictated by the task would then be appropriate.

#### Interpretability and robustness in application will require method development.

Each instrument configuration will slightly change the data recorded, which can now be rigorously predicted by theory (136). Though instrumentation can vary, high accuracy can be obtained for most current configurations (137). However, the issue of robustness of prediction algorithms across instrument configurations is not fully settled (138). The SNR of recorded data influences classification results, but there is likely a limit for most applications in which further increases in accuracy are not possible by increasing the SNR (67). For the sake of stability of the results, this limit should be found for each workflow and the data checks implemented to ensure the SNR is sufficiently high. While significant work has focused on the batch effect in histopathology with DL, the small number of configurations and the need for the development of workflows for each application imply that this issue is not likely to be an active area. Given the still-early stage of application development, demonstration of robustness of algorithms for a complete solution (instrument, analysis and visualization) for multiple clinical settings, demonstration of robustness in operation by nonexpert users, integration with clinical workflows, and more applications are likely to dominate activities in the near future.

#### Increasing chemical detail and precision of spectroscopic imaging will be possible with a combination of physics-based modeling and DL.

Tissues are well known to have multiscale organization. At the molecular level, chirality may be apparent, subcellular organization is influenced by function (e.g., cytoskeleton), cellular organization is often dictated by organ functions and determines tissue architecture (e.g., apical and basal organization of epithelial cells), and the tissue matrix often involves molecular to multilength scale physical orientation (e.g., collagen bundles in the stroma). In addition to advances in instrumentation, computational algorithms must be harnessed if this additional information is to be used in histopathology. Since the additional data needs more acquisition time, one goal is to speed up information recovery. Another emerging goal is to use the additional information to correct sample-dependent distortions in recorded data, recovering the morphologic structure and spectra simultaneously. Though IR spectroscopic imaging is often described as a straightforward combination of IR spectroscopy’s molecular selectivity and optical microscopy’s spatial specificity, our understanding of the recorded data is not as straightforward. The complex interplay of sample structure, beam effects arising from coherence, thermal lensing, and the impact of changing optical configurations might all be amenable to DL treatments. However, a significant database of samples and applications must be built first.

## PATHOLOGY WORKFLOWS AND TRANSLATION: OUTLOOK

With large areas being imaged rapidly (139), IR imaging is showing signs of considerably shortening and integrating workflows for pathology by eliminating the need for staining, automating microscopy, and integrating knowledge extraction. However, the burden of proof and the barrier to entry for all applications are not equal. One knowledge gap is the lack of a relationship between the complexity of the problem and the evidence needed for successful IR imaging. At this emergent state of the field, it is important to categorize studies, as well as choices from the diversity of instrumentation and methods developed in broad categories of use, so that they can be systematically understood and developed for pathology.

### Categories for Applications of IR Imaging in Pathology

While it is not possible to delineate each use case, this review suggests a broad categorization (Table 2) and provides several examples to illustrate commonalities within the categories. Category I includes applications to identify cells that are cumbersome by traditional techniques [e.g., high-definition profiling of lymph nodes for all cells (140) or providing specific biochemical insights such as metabolism in glioblastoma cells (141)]. Stain-free identifications can also be used to condition accurate and detailed molecular analyses (e.g., with mass spectrometry) (142). Category II applications include the use of chemical information to recognize disease or physiologic states within specific cells. Tissue fibrosis, for example, is often associated with disease progression and is detectable (143) in the manner of immunofluorescence microscopy (144). Measuring the fibrotic reaction highlights the biochemical nature of processes [e.g., between liver diseases and diabetes (145)] and reduces the effort needed to obtain molecularly specific results (146). Category III applications use chemical and spatial information to provide objective and automated subclasses of disease (e.g., grade) that can aid or confirm clinical diagnoses [e.g., sub-classification of lung cancer (147) or prostate cancer grading and staging (148)]. In Category IV applications, chemical imaging data can be used for prognostic or predictive applications, for example, to deduce the spatial-chemical syntax of prostate tumors to define potentially recurrent ones (149) or to assess moderate-grade colon tumors to determine more aggressive ones (68). Many of these applications highlight that IR imaging can measure all cells simultaneously and some disease-associated changes in each cell as well. This capability can allow researchers to study disease processes based on the microenvironment, adding more information than is currently available. This ability for comprehensive analysis also makes spatial coverage an important consideration. WSI is typical in optical microscopy, in which slides are scanned in minutes. Although IR images smaller than 1 × 1 mm are typical today, greater integration with pathology will require the typical standard for WSI (15 × 15 mm) to be measured in less than 10 min.

### Guidelines for Reporting Studies Using IR Imaging in Pathology

The design of studies, the performance needed for instrumentation, the complexity of algorithms, and the strength of the evidence are all dependent on the complexity and importance of each category listed above. With the categorization of applications, a coalesced set of guidelines for studies (Table 3), termed experimental and analytical specifications for spectroscopic imaging in histopathology (EASSI-H), can also be proposed. Although not every recommendation may be applicable to each study, the hope of this proposal is to provide practitioners with a checklist of information that should be included to provide readers with a complete picture of the study. With clear explanations of the work done, and with access to data and facilitation of interstudy comparisons, adopting these guidelines can help accelerate progress with better communication and a common platform to judge the strength of the evidence in the context of the study design and claims of its success.

### The Potential Impact of IR Imaging in Pathology

Introducing IR images and the visualizations they enable can greatly reduce the focus in pathology from hunting for occurrences of disease or specific cells to interpreting the data. This could be a paradigm shift enabled by IR spectroscopy. It is natural to expect digital methods applied to conventional pathology images to precede the use of IR imaging. Driven by advances in technology, approvals by the US Food and Drug Administration, development of workflows for many use cases, and a general acceptance of cyber resources for (remote) work, digital methods have now become the primary diagnostic tools at some organizations (150). In the opinion of the author, the paramount challenge for IR imaging today is demonstrating utility. Before undertaking translation, a specific and practical workflow needs to be developed for a use case that can be standardized, and the reproducibility of this workflow within and between clinical settings as well as over time needs to be carefully demonstrated. Each workflow should be suited to a specific problem and its accuracy needs to be validated in a clinical context with multiple independent cohorts of samples and operations in actual use settings. Currently, no developments meet these standards. Yet another step that needs to be proven is that the research or clinical utility of IR imaging must make activities more productive. In research, this might imply more information, fewer resources, or more accuracy for assays. For clinical diagnostics, these methods should improve decision-making compared with extant methods alone and lead to better patient outcomes. Several groups are focusing on innovative solutions to clinical pain points, and development of integrated hardware-software protocols that are easy to use and cost-effective are also needed. New technologies based on IR imaging will replace the common microscope and extant methods only if the knowledge they yield is more valuable and more cost-effective than the state of the art. IR imaging has now matured to the extent that contemplating a radical change in diagnostic and research pathology is possible by this simple and integrative means to record relevant data, parse this knowledge, and make it useful. With many advantages, IR imaging offers a potential route to add to and even replace some of the methods that have been prevalent for decades.

## Figures and Tables

**Table 1:** Characteristics of continuous and DFIR imaging systems

	Continuous spectra imaging data	DFIR chemical images
**Measurement**	Broadband spectrum, uniform spectral noise, and resolution	Narrowband, independent properties of spectral bands
**Spectral resolution mechanism**	Time series measurement (i.e., interferometer scanning)	Narrowband snapshot (e.g., grating position)
**SNR advantages**	Spectral multiplexing, throughput	High source intensity
**Defining characteristic**	High wavelength fidelity, high SNR for weak sources	High signal, independent modulation capability
**Most useful for**	Exploratory analyses, discovery	Fit-for-purpose histopathology
**Imaging constraints**	Reflective optics needed, cumbersome for hyphenated techniques	Beam coherence, suited for hyphenated techniques

Abbreviations: DFIR, discrete frequency infrared; SNR, signal-to-noise ratio.

**Table 2:** A proposed categorization of applications for spectroscopic imaging in histopathology

Category of applications	Characteristics			
	Goal(s)	Typical design	Data characteristics	Applications
**Category I: Identification of tissue composition**	Identify cells and constituents (i.e., pixel-level features) Identify cell types in tissue from compositional differences Measure biochemical composition or heterogeneity Detect extracellular components	Many spectral instances (pixels) available within a single sample	Spectral differences between cells likely larger than diversity in populations Large spectral differences are expected and relatively simple Analyses are often univariate and are effective	Well-established approach Many examples available (e.g., histology on many tissue types, microcalcifications, detection of disseminated tumor in lymph nodes) Output may serve as input for downstream analyses (e.g., with laser capture microdissection)
**Category II: Identification of disease, physiology, or development**	Identify disease, differences in physiology, or function in a sample (i.e., regional features)	Spectral instances include both pixels and whole regions (e.g., tumors in ducts) Many examples and patients available	Subtle chemical differences Often affected by sample preparation Requires more sophisticated algorithms and careful spectral processing	Detection of disease and heterogeneity within disease Detection of microenvironmental changes associated with disease progression Prediction of molecular expression Validation with IHC
**Category III: Disease characterization**	Determine severity of disease or subclasses that are clinically relevant (i.e., sample-level features)	Spectral instances and heterogeneity of tissue may need to be considered Determination at the patient level Examples and patients may be available for common diseases but heterogeneity measures and new classes may require prospective studies	Subtle chemical differences often need to be considered in a spatial context May require multimodal information Requires significantly sophisticated algorithms and larger validation effort Unambiguous ground truth (e.g., disease grade) may be difficult to obtain	Recapitulation of disease grades Discovery of subclasses of disease
**Category IV: Prognostic and predictive, individualized analyses**	Prognostication (outcome, regardless of therapy) and prediction (effect of a therapeutic intervention), with an ultimate goal of individualized results (i.e., human-level features)	Complete IR information and clinical and other information are incorporated into models for patient-level predictions New information from algorithms may be used to guide searches for biological causes of observed predictions Need to relate output of algorithms to images	Multiple tissue components and spectral changes considered in a spatial context Often requires clinical and multimodal information Requires significantly sophisticated algorithms Retrospective validation combined with prospective validation is highly desirable	Prognostication and prediction More individualized (group) and precise (patient-level) information that is often clinically actionable

Abbreviations: IHC, immunohistochemical; IR, infrared.

**Table 3:** Proposed experimental and analytical specifications for spectroscopic imaging studies in histopathology

Category	Essential specifications
Patient and clinical data	Characteristics of patients selected (disease, control) Source of material Demographics of patients (e.g., age, sex, ethnicity)
Sample processing	Sample history from acquisition to measurement Processing steps used Preservation and storage conditions Pathology reference slides (e.g., H&E or IHC stains) Quality assessment processes (e.g., pathologist review)
Study parameters	Goal of the study Inclusion/exclusion criteria Control samples Case selection (e.g., prospective or retrospective, stratification or matching) Target statistics (e.g., power)
Specimen preparation	Substrate used Protocol to cut and deposit Chemical treatments (e.g., hexane wash for 24 h)
Measurement conditions	Description of optical setup Detailed measurement protocol Quality reference and data (e.g., standard spectrum, 1951 USAF resolution test-equivalent sample) Reproducibility checks for instrument
Data processing	Description of all preprocessing steps (e.g., baseline correction, normalization derivatives, smoothing) Spectral and other variables considered and selected (with selection algorithm) SNR calculation method and SNR evaluation of data
Machine learning	Model selection and alternatives considered or rejected during study Algorithm Full code and parameters Sample sets for training, testing, and validating steps Missing information/unbalanced data handling
Claims	Relation to ground truth Statistical measures of results including sample size Quantitative measures (e.g., ROC curves, Kaplan-Meier plots, hazard ratios) with confidence intervals Precise description of end points Model verification
Study assessment	Relationship between spectral data and diagnostic test performance Sensitivity analysis Limitations of the study Comparison with clinical/research standard

Abbreviations: H&E, hematoxylin and eosin; IHC, immunohistochemical; ROC, receiver operating characteristic; SNR, signal-to-noise ratio; USAF, United States Air Force.

## SUMMARY POINTS

1. Infrared (IR) spectroscopic imaging combines the molecular selectivity of mid-IR vibrational spectroscopy and the spatial specificity of optical microscopy.
2. Application of IR imaging to pathology can simplify workflows by eliminating the need for staining and by providing informative, color-coded images that can be used for decision-making.
3. IR spectroscopic imaging is a rapidly evolving technology, with several variants providing high SNR, high resolution, and expanded molecular information, including orientation and chiral composition.
4. Machine learning, including deep learning (DL), is necessary and powerful for extracting information from spectra, and emerging approaches can harness spectral-spatial correlations as well.
5. One of the most fruitful areas of application will be to reframe the prevalent view of cancer, from a mass of cells of uncontrolled proliferation to a complex society of cells (e.g., stromal changes, blood vessels, and immune cells) that compose the microenvironment.
6. Four categories of applications, with increasing complexity and analytical demands, are proposed for IR imaging.
7. Guidelines for studies reporting the use of IR imaging for histopathology are proposed.

## FUTURE ISSUES

1. Technology development for IR imaging has greatly accelerated, and further increases in speed, resolution, and chemical detail will lead to new applications.
2. Practical technology that can be incorporated into existing diagnostic or research workflows is needed.
3. Digital pathology methods are becoming more accepted, and additional useful information and ease of use of IR imaging will need to be demonstrated.
4. DL offers a powerful means to leverage both spatial and structural information from IR imaging and will be a significant forum for enabling its use for pathology.
5. Utility of IR imaging to solve clinical problems is the starting step for translation, and use cases need to be developed.
